# Long-term fertilization determines different metabolomic profiles and responses in saplings of three rainforest tree species with different adult canopy position

**DOI:** 10.1371/journal.pone.0177030

**Published:** 2017-05-11

**Authors:** Albert Gargallo-Garriga, S. Joseph Wright, Jordi Sardans, Míriam Pérez-Trujillo, Michal Oravec, Kristýna Večeřová, Otmar Urban, Marcos Fernández-Martínez, Teodor Parella, Josep Peñuelas

**Affiliations:** 1 CSIC, Global Ecology Unit CREAF-CEAB-CSIC-UAB, Cerdanyola del Vallès, Catalonia, Spain; 2 CREAF, Cerdanyola del Vallès, Catalonia, Spain; 3 Servei de Ressonància Magnètica Nuclear, Faculty of Sciences and Biosciences, Universitat Autònoma de Barcelona, Bellaterra, Barcelona, Catalonia, Spain; 4 Smithsonian Tropical Research Institute, Apartado, Balboa, Republic of Panama; 5 Global Change Research Institute, The Czech Academy of Sciences, Brno, Czech Republic; Austrian Federal Research Centre for Forests BFW, AUSTRIA

## Abstract

**Background:**

Tropical rainforests are frequently limited by soil nutrient availability. However, the response of the metabolic phenotypic plasticity of trees to an increase of soil nutrient availabilities is poorly understood. We expected that increases in the ability of a nutrient that limits some plant processes should be detected by corresponding changes in plant metabolome profile related to such processes.

**Methodology/Principal findings:**

We studied the foliar metabolome of saplings of three abundant tree species in a 15 year field NPK fertilization experiment in a Panamanian rainforest. The largest differences were among species and explained 75% of overall metabolome variation. The saplings of the large canopy species, *Tetragastris panamensis*, had the lowest concentrations of all identified amino acids and the highest concentrations of most identified secondary compounds. The saplings of the “*mid canopy”* species, *Alseis blackiana*, had the highest concentrations of amino acids coming from the biosynthesis pathways of glycerate-3P, oxaloacetate and α-ketoglutarate, and the saplings of the low canopy species, *Heisteria concinna*, had the highest concentrations of amino acids coming from the pyruvate synthesis pathways.

**Conclusions/Significance:**

The changes in metabolome provided strong evidence that different nutrients limit different species in different ways. With increasing P availability, the two canopy species shifted their metabolome towards larger investment in protection mechanisms, whereas with increasing N availability, the sub-canopy species increased its primary metabolism. The results highlighted the proportional distinct use of different nutrients by different species and the resulting different metabolome profiles in this high diversity community are consistent with the ecological niche theory.

## Introduction

Nutrient limitation has been widely observed in forests at a global scale [1]. The balance of nutrient supplies, losses and concentrations change as soils age [2,3]. The weathering of bedrock is the primary source of P and K, and their availability tends to decline as soils age [2,4]. In contrast, N is largely absent in most rocks, its primary sources are N fixation and atmospheric N deposition, and its availability increases with time, reaching a maximum in moderately leached soils, and declining in highly leached soils [5,6]. Meta-analyses of data from fertilization experiments have shown that N, P and K each limit a large percentage of forest plant species [7–11]. However, although some authors such as LeBauer and Treseder (2008) did give a substantial amount of attention to lowland tropical forests, in general these forests have received only limited attention.

For example, several studies that took place at a long term fertilization experiment in Panama have corroborated that N, P and K each co-limit forest plant function [12–14]. The addition of K reduced fine root biomass, increased fine root turnover, decreased seedling root-to-shoot ratios, increased stomatal conductance, increased herbivory, and increased growth rates of seedlings. The addition of P decreased soil phosphatase activity, and increased soil microbial biomass, root arbuscular mycorrhizal infestation, photosynthesis, herbivory, and fine litter production [12,14–18]. N addition acidified the soil by 0.8 pH, decreased root arbuscular mycorrhizal infection, increased photosynthesis, and, when added with K, increased growth rates of saplings and poles [12,19,20]. The responses to fertilization were species-dependent. The species that grow at different canopy levels respond differently in their eco-physiological variables and growth to different nutrient fertilization [13,21].

The recently proposed biogeochemical niche hypothesis [22–24] claims that each species has an optimal elemental composition and stoichiometry as a result of its optimal function in its specific ecological biogeochemical niche. This optimal elemental composition results from the differences in functions and morphologies, developed over a long period of time resulting in each species tending to reach an optimum chemical composition linked to a singular optimum function (homeostasis). In addition, plant species should have, to some degree, a flexible adaptation capacity to alter their elemental stoichiometries in response to changes in the composition of neighboring species and/or in environmental conditions (such as climate gradients) [25–27]. Species exhibit a certain degree of stoichiometric flexibility to be able to respond to environmental changes and competition, probably with a tradeoff between adaptive capacity (flexibility) and stability (homeostasis) [28]. Thus, we hypothesized that different sympatric species coexisting in the rainforest would use nutrients in different proportion given their specialization in a determined niche and would respond differently to the increase of different nutrient availabilities to avoid competition among them. Thus, we expected that the genotypic differences that confer distinct growth “programme” should imply a different use of the nutrients supplied by fertilization with changes in the metabolic function among them. We also expected that when the availability of a limiting nutrient increases the metabolic pathways related to growth such as primary metabolites (aa or nucleotides) should be enhanced leading to higher ARN concentrations and higher protein synthesis. N and P are necessary to build proteins (aa) and DNA and RNA and P to build DNA and RNA. If these nutrients are in low availability then the metabolism related to aa synthesis is down-regulated, whereas it is upregulated if the availability increases. The stress caused by nutrient limitation can also favour the up-regulation of anti-stress secondary metabolism with more C-rich metabolites.

The metabolome of an organism is known to be highly responsive to internal and external stressors [29–32], being considered the final expression of an organism’s genotype at a particular moment [30,33,34]. Thus, the analysis of the differences in overall foliar metabolic and molecular function among sympatric species of tropical rain-forests growing under different nutrient availabilities as a result of long-term fertilization would be of great importance for a better understanding of the species-specific functional differences and responses to different nutrient availabilities. The up and down regulation of different metabolic pathways, from those of primary metabolites such as sugars or amino acids to those of secondary metabolites such as phenolics or terpenes, should provide a more solid understanding of how nutrients are differently used by distinct species in a primary tropical rainforest.

We hypothesized that each one of these tree species would have different metabolomic profiles and also different metabolomic responses to the fertilization in accordance with their long-term adaptation to differential use of different resources in distinct ecological niches such as those defined by distinct canopy position. Thus, we aimed to use metabolomics analytical tool to obtain information on the long-term impacts of these chronic fertilization treatments on the overall function of different tree species that coexist in the same tropical forest. As a consequence the changes in nutrient availabilities should affect differently the metabolomic structure of the distinct species with metabolic pathways up- and/or down regulated depending on particular species. The knowledge of distinct metabolomic shifts in different species in response to chronic fertilization would support the hypothesis of the different proportional use of the diverse nutrients by sympatric species in this high-diverse ecosystem. We have conducted an ecometabolomic profile study in the Gigante fertilization experiment of Panama by studying three of the most abundant tree species, *Tetragastris panamensis*, *Alseis blackiana* and *Heisteria concinna* growing in the NPK fertilization experiment [11]. Thus, we expected that each one of these tree species would present different metabolomic profiles and also different metabolomic responses to the fertilization in accordance with their long term adaptation to differential use of different resources in different ecological niches, and also in accordance with the evolutionary pressure to avoid competition in these tropical forests.

## Materials and methods

S. Joseph Wright, Senior staff of the Smithsonian Tropical Research Institute, has studied the site for many years now as person in charge of the research of the fertilization plots. We confirm that field studies did not involve endangered of protected species. No specific permissions were required.

### Field site

The 38.4-ha study site (9° 06’ 31” N, 79° 50’ 37” W) is located on the mainland in the Barro Colorado Nature Monument in the Republic of Panama. Tree species composition and stature (canopy heights up to 40 m) are characteristic of very old (>200yr), secondary forest. The bedrock is an 85-m thick Miocene basalt and is relatively poor in P [12]. For further site description see Wright et al. (2011) and Yavitt et al. (2009).

### Field experiment and sampling

**Fig 1:**
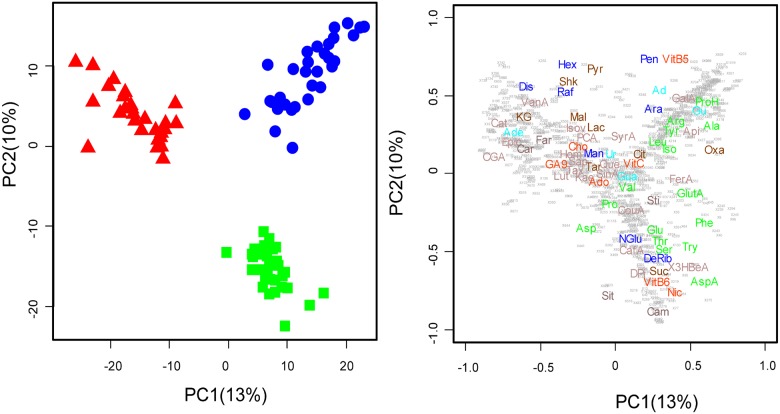
Principal component analyses (PCAs) of metabolomic data illustrating the samples (cases) belonging to different species (panel A) and variables (panel B; only the elucidated molecules are depicted). Species are indicated by different colors and shapes (green squares, *Alseis blackiana*; red triangles, *Tetragastris panamensis*; blue circles, *Heisteria concinna*). Organic acids (Orange): Tar;Tartaric acid, Cit;Citric acid (monohydrate), KG;α-Ketoglutaric acid, Lac;Lactic acid, Mal;L-(-)-Malic acid, Oxa;Oxalacetic acid, Pyr;Pyruvic acid, Shk;Shikimic acid, Suc;Succinic acid, Aminoacids (green): Ala;Alanine, Arg;Arginine, Asp;Asparagine, AspA;Aspartic acid, Glu;Glutamine, GlutA;Glutamic acid, Iso;Isoleucine, Leu;Leucine, Phe;Phenilalanine, Pro;Proline, ProH;Hydroxy-rolin, Ser;Serine, Thr;Threonine, Try;Trypthofan, Tyr;Tyrosine, Val;Valine, Phenolics Compounds (lilac): Api;Apigenine, CafA;Caffeic acid, Cat;DL-catechin, CGA;Chlorogenic acid, CouA;3-coumaric acid, DPi;D-Pinitol, Epic;(-)-epicatechin, FerA;Ferulic acid, GalA;Gallic acid, Hom;Homoorientin, Isov;Isovitexin, Kae;Kaempferol, Lut;Luteolin, PCA;protocatechuic acid, Que;Quercetin, Sap;Saponarin, SinA;Sinapic acid, SyrA;Syringaldehyde, Tax;Taxifolin, 3HBeA;3-hydroxybenzoic acid, VanA;Vanillic acid, Bases Nitrogen (light blue): Ad;Adenine, Ade;Adenosine, Gu;Guanine, Gua;Guanosine, Ur;Uracil, Terpenes (Brown): Cam;campesterol, Car;Carvone, Far;Farnesol, Sit;sitosterol, Sti;stigmasterol, Sugars (blue);, Ara;D-(+)-Arabitol+Xylitol, DeRib;2-Deoxy-D-ribose—fucose, rhamnosa, Dis;disacharide, Hex;hexose, Man;SumaD-Mannitol,Dulcitol,Sorbitol, NGlu;N-acetylD-glucosamine, Pen;pentosy, Raf;D-(+)-Raffinosepentahydrate(raffinose+maltotriose+melezitose), Others: Ado;Adonitol (Ribitol), Cho;Choline, GA9;Gibberellic acid (GA9), Nic;Nicotine, VitB5;Pantothenic acid (Vit B5), VitB6;Pyridoxine (Vit B6), VitC;Ascorbic acid (Vit C).

The 32 experimental plots measured 40 x 40 m each one. The minimum distance between plots was 40 m, except for two plots that were separated by 20 m and a 3 m deep streambed. The experiment was established in 1998 and consisted of eight treatments of a 2 x 2 x 2 factorial NPK experiment four times. Four replicates were placed perpendicular to the 36-m topographic gradient because soil properties [35] and tree distributions (S. J. Wright, unpublished data) paralleled the gradient. Within each replicate, the N, P, K, and NPK treatments vs. the NP, NK, PK, and control treatments were blocked (Fig 1). This balanced, incomplete-block design minimizes uncontrolled error associated with spatial variation, enables evaluation of main effects and two-way interactions, but limits power to evaluate the three-way interaction [36].

Beginning in 1998, we added fertilizer by hand in four equal doses each wet season with 6–8 weeks between applications (15–30 May, 1–15 July, 1–15 September, and 15–30 October). Nitrogen was added as coated urea ((NH_2_)_2_CO), P as triple superphosphate (Ca(H_2_PO_4_)_2_.H_2_O), and K as potassium chloride (KCl). Annual doses were 125 kg N ha^-1^yr^-1^, 50 kg P ha^-1^yr^-1^, and 50 kg K ha^-1^yr^-1^, which equals 69%, 470%, and 88% of annual inputs from fine litter at a nearby site (3 km), respectively (Table 1). Similar large additions of P relative to annual litter inputs are standard practice in forestry and in previous tropical nutrient addition experiments [37–39] because many soils, including soils at our site, sequester large amounts of added P in forms that are inaccessible to plants [19]. After nine years, N addition had reduced soil pH and base saturation and increased nitrate leaching, N-oxide emissions, and aluminum saturation [40].

**Table 1:** PERMANOVA for the whole metabolite data set after Hellinger transformation for each species. Bold type indicates significant effects (*P* < 0.05). Italics type indicates marginally significant effects (*P* < 0.1).

Independent variables	Df	Sums of squares	Mean squares	F. Model	*R*^*2*^	*P*
**N**	**1**	**0.1845**	**0.184484**	**3.9056**	**0.03979**	**< 0.0001**
**K**	1	0.0514	0.051361	1.0873	0.01108	0.32
**P**	**1**	**0.2201**	**0.220129**	**4.6602**	**0.04747**	**< 0.0001**
***N*:*K***	*1*	*0*.*0734*	*0*.*073357*	*1*.*553*	*0*.*01582*	*0*.*061*
**N:P**	**1**	**0.1162**	**0.116154**	**2.459**	**0.02505**	**0.002**
***K*:*P***	*1*	*0*.*0708*	*0*.*070774*	*1*.*4983*	*0*.*01526*	*0*.*073*
**Residuals**	83	3.9206	0.047236		0.84553	
**Total**	89	4.6368			1	

Leaf samples were collected from shaded understory saplings and immediately frozen in liquid N in the field. Sampled species were *Tetragastris panamensis* (Engler) Kuntze (Burseraceae), *Alseis blackiana* Hemsl. (Rubiaceae) and *Heisteria concinna* Standl. (Olacaceae). We sampled one individual of each tree species in each one of the 32 experimental plots with four exceptions. *Tetragastris panamensis* was absent from one +P, one +PK and one +NPK plot and *Alseis blackiana* was absent from one +P plot. The trees were randomly selected from the available trees between 1 cm and 10 cm diameter breast height in each plot. Leaves were between 1 and 2.5 m above ground. We randomly collected 4 g fresh mass i.e. ca. 8 leaves of *Alseis blackiana*, 6 leaves of *Heisteria concinna*, and 2 leaves of *Tetragastris panamensis* receiving 1.5% to 3% of full sunlight with most around 1.5%.

### Liquid chromatography-mass spectrometry (LC-MS) analysis

We performed a metabolomics analysis using the methodology described in Gargallo-Garriga *et al*., 2014. Briefly, LC-MS chromatograms were obtained using an Ultimate 3000 high performance liquid chromatography system (Dionex, Waltham, Massachusetts, USA) coupled to an LTQ Orbitrap XL high-resolution mass spectrometer (Thermo Fisher Scientific, Waltham, Massachusetts, USA) equipped with an HESI II (heated electrospray ionisation) source. Chromatography was performed on a reversed-phase C18 Hypersil gold column (150 × 2.1 mm, 3-μ particle size; Thermo Scientific, USA) at 30°C. The mobile phases consisted of water (0.1% acetic acid) and acetonitrile. For the preparation of the sample, 150 mg of plant were weighted. The electric signal from LC-MS of the spectrometers is used since it is directly related to the concentration of the molecular compound.

## Statistical analyses

To avoid species bias in the analysis of the effects of fertilization treatments, we standardized the data using the Hellinger transformation *y*_*ij*_*’* = (*y*_*ij*_ / *y*_*i+*_)^^0.5^, where *y*_*ij*_ is the value determined for species *j* in treatment *i*, *y*_*i+*_ is the sum of the values over all species for treatment *i*, and *y*_*ij*_*’* is the Hellinger transformed value. We performed eight different analyses with the Hellinger transformed values. The full dataset with all available is provided in S1 Table.

First, we performed four PERMANOVA analyses with Euclidian distances in which the response term was the metabolite data matrix and the predictor variables were the eight factorial nutrient treatments (control, N, K, P, NK, NP, KP, NPK). In the first PERMANOVA analysis, we used all three species together and included the species as a predictor. We also performed PERMANOVA analyses for each species separately. There were no significant species-treatment interactions (Table 1). Therefore, we transformed values for all species to a common scale through Hellinger transformation and dropped the species main and species interaction effects on the metabolome profile. We then repeated the PERMANOVA to evaluate nutrient effects and their two-way interactions (N*K, N*P and P*K). In this case there was a significant effect of N and P fertilization, but not of K fertilization (Table 1). The N*P interaction was also significant (Table 1).

Second, we performed four multivariate ordination analyses. We performed a principal component analysis (PCA) used to discriminate species. We performed a sparse partial least squares (sPLS) to evaluate the relationships between experimental treatments and metabolites. We performed a one-way ANOVA based on scores for the first component of a partial least squares discriminant analysis (PLS-DA). In the one-way ANOVA, the fertilization treatments comprised the single categorical independent variable, with eight levels corresponding to the eight factorial combinations of N, P and K. We used post-hoc Tukey HSD tests to identify treatments that differed significantly.

Our final analysis consisted of clustered image maps (CIM). The CIM representation is based on a hierarchical clustering of the 50 metabolites that best separated the fertilization treatments in the first three dimensions (axes) of the sPLS. The hierarchical clustering was based on a pair-wise similarity matrix obtained from the sPLS. The values in the similarity matrix are a robust approximation of the Pearson correlation (see in more detail González, et al., 2012). Euclidian distance and the Ward method were used for the hierarchical clustering. In the CIM display, each coloured block represents an association between subsets of the independent variables (fertilization treatments) and the response variables (metabolites). The relationships between the studied independent categorical variables (fertilization and species) and their interaction on each metabolic signal and on the scores of each case in the multivariate analyses (PCA, PLS-DA, sPLS) were conducted by a factorial ANOVA with Bonferroni post hoc test.

A Kolmogorov-Smirnov (KS) test was performed for each metabolite to evaluate normality. The analytical signals of all detected metabolites (identified and unidentified) were normally distributed and with homogeneous variance. All analyses were conducted with R (R Development Core Team 2008). The multivariate ordinations were conducted with the *mixOmics* package of R. For more detailed explanation of data analysis see Gargallo-Garriga *et al*. 2015, 2016 [41,42].

## Results

### PERMANOVA analyses

In the PERMANOVAS conducted for each species separately, *T*. *panamensis* was most sensitive to nutrient availability. There were significant effects of K and P in *T*. *panamensis*, marginally significant effects of N in *A*. *blackiana*, and no significant nutrient effects in *H*. *concinna* (Table 2).

**Table 2:** PERMANOVAs for the metabolite data set of all species. Bold type indicates significant effects (*P* < 0.05). Italics type indicates marginally significant effects (*P* < 0.1).

*A*. *blackiana*						
**Independent variables**	**Df**	**Sums of squares**	**Mean squares**	**F. Model**	***R***^***2***^	***P***
***N***	*1*	*0*.*06555*	*0*.*065548*	*1*.*88708*	*0*.*06315*	*0*.*066*
**K**	1	0.02238	0.022379	0.64428	0.02156	0.77
**P**	1	0.05341	0.05341	1.53762	0.05145	0.14
**N:K**	1	0.02166	0.021656	0.62345	0.02086	0.79
**N:P**	1	0.02477	0.02477	0.71312	0.02386	0.71
**K:P**	1	0.01663	0.016633	0.47884	0.01602	0.90
**Residuals**	24	0.83365	0.034735		0.80309	
**Total**	30	1.03804			1	
*H*. *concinna*						
**Independent variables**	**Df**	**Sums of squares**	**Mean squares**	**F. Model**	***R***^***2***^	***P***
**N**	1	0.02222	0.022217	0.47982	0.01539	0.69
**K**	1	0.02905	0.029046	0.62731	0.02012	0.57
**P**	1	0.03259	0.032594	0.70394	0.02257	0.51
**N:K**	1	0.06323	0.063227	1.36552	0.04379	0.19
**N:P**	1	0.08151	0.081508	1.76032	0.05645	0.11
**K:P**	1	0.05782	0.057825	1.24884	0.04005	0.22
**Residuals**	25	1.15757	0.046303		0.80165	
**Total**	31	1.44399			1	
*T*. *panamensis*						
**Independent variables**	**Df**	**Sums of squares**	**Mean squares**	**F. Model**	***R***^***2***^	***P***
**N**	1	0.04818	0.048181	1.08693	0.04086	0.33
**K**	**1**	**0.0976**	**0.0976**	**2.20179**	**0.08277**	**0.022**
**P**	**1**	**0.09381**	**0.09381**	**2.11629**	**0.07955**	**0.020**
**N:K**	1	0.01992	0.019923	0.44944	0.01689	0.89
**N:P**	1	0.01567	0.015669	0.35348	0.01329	0.94
**K:P**	1	0.01749	0.017488	0.39451	0.01483	0.92
**Residuals**	20	0.88655	0.044328		0.75181	
**Total**	26	1.17922			1	

### Multivariate ordination analyses

#### Species effects

The principal component analysis (PCA) separated the species along the biplot of the first two principal PC axes, which together explained 23% of the total variance (Fig 1). The most significant differences in metabolic profile were between *T*. *panamensis* and *A*. *blackiana*, which were separated along the first PC axis (Fig 1). Metabolomes of *T*. *panamensis* were associated with the highest concentrations of shikimic acid, pyruvate, malate, lactate, most identified secondary compounds, and phenolics and the lowest concentrations of most identified amino acids with respect to the other two species (Fig 1 and S2 Table). In contrast, *H*. *concinna* metabolomes were associated with the highest concentrations of several amino acids, and *A*. *blackiana* metabolomes were associated with the highest concentrations of other amino acids and phytosterols (Fig 1 and S2 Table).

#### Treatment effects

The one way ANOVAs conducted with the scores of partial least squares discriminant analysis (PLS-DA) as the dependent variable and the fertilization treatments as the independent variable showed a significant difference among the eight fertilization treatments (S3 Table). Two other post hoc pairwise comparisons of fertilization treatment, N vs K and N vs PK showed different metabolome profiles (S3 Table). The post-hoc tests for the scores of component 1 of the PLS-DA showed that the significant differences among fertilization treatments were almost entirely due to highly significant differences between the control treatment and six of the seven fertilization treatments (S2 Table).

**Fig 2:**
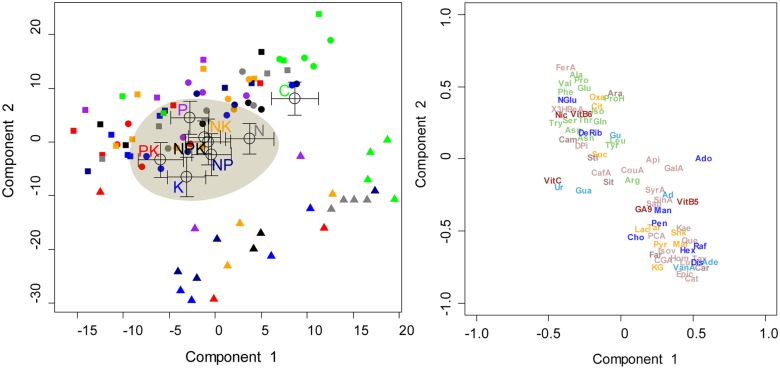
Biplots of the two first components of the PLS-DA of the Hellinger transformed metabolomics data presenting the scores (mean ± S.E.) of the different fertilization treatments (N, P, K, NP, NK, PK, NPK and C). Species are indicated by different shapes (squares, *Alseis blackiana*; triangles, *Tetragastris panamensis*; circles, *Heisteria concinna*) and fertilization treatments by colours (grey, N; purple, P; blue, K; dark blue, NP; yellow, NK; red, PK; dark, NPK; green, C). The metabolomics variables distribution (only the elucidated molecules are depicted). Compounds as in Fig 1.

When Hellinger transformed values were pooled for the three species, component 1 of the partial least squares discriminant analysis (PLS-DA) separated the control treatment from all fertilized treatments except the N fertilized treatment (Fig 2). Among the identified metabolites, higher concentrations of most amino acids, vit C, vit B6 and nicotinic acid were observed in fertilized trees. Component 2 separated the metabolome profiles of the control treatment from all fertilized treatments except the P fertilized treatment. In the fertilized and control trees sugars, terpenes and phenolic concentrations were most clearly linked with these differences (Fig 2).

**Fig 3:**
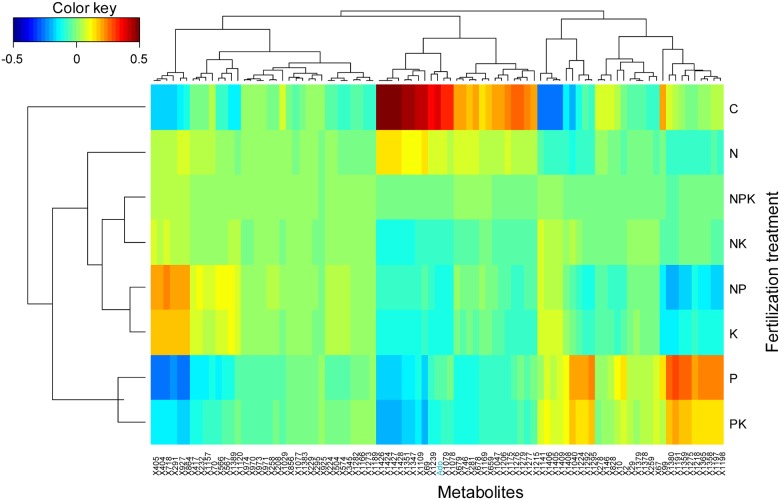
Clustered image maps of the metabolites in different fertilization treatments based on the data of the PLS analysis with the Hellinger transformed metabolomics data. The red and blue colors indicate positive and negative correlations respectively.

We performed a cluster image map with the metabolites whose concentrations differed significantly among the fertilization treatments. The results showed a cluster of 11 non-identified metabolites with higher concentrations in control trees with respect to all the fertilization treatments, a cluster of 6 non-identified metabolites with highest concentrations in K and NP fertilized trees and lowest concentrations in P fertilized trees, and a cluster of 9 non-identified metabolites with highest concentrations in P and PK fertilized trees (Fig 3).

As observed in the PCA analysis (Fig 1), the differences among species were very large and hid the smaller intraspecific differences induced by fertilization. However, when we conducted a PLS-DA with Hellinger transformed values and included all three species, we observed that control trees were significantly separated from trees in the N, P NK and NPK treatments (Fig 2).

**Fig 4:**
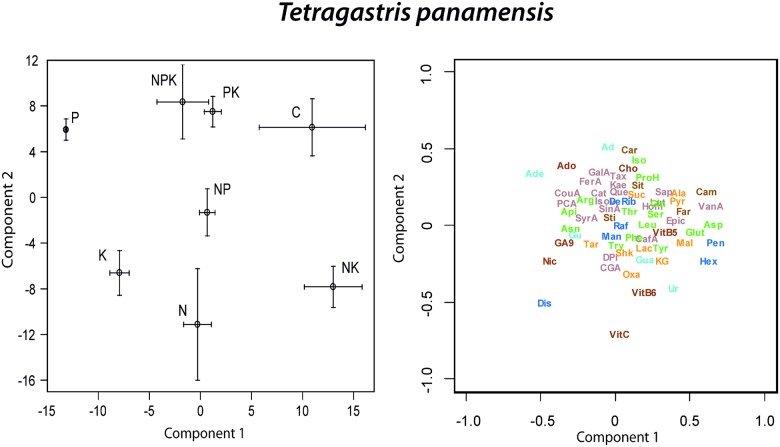
Biplots of the two first factors of the PLS-DA of *Tetragastris panamensis* metabolomics data presenting the mean scores (± 1 S.E.) of the different fertilization treatments (N, P, K, NP, NK, PK, NPK and C) and the metabolomics variables distribution (only the elucidated molecules are depicted). Compounds as in Fig 1.

**Fig 5:**
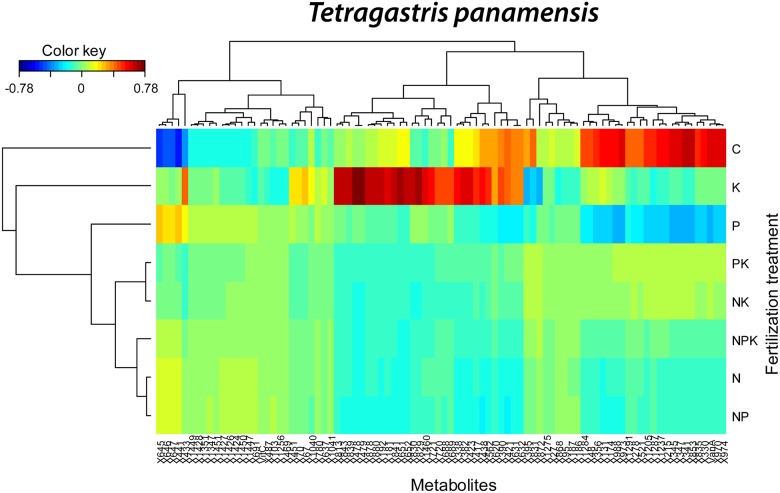
Clustered image maps of the metabolites of *Tetragastris panamensis* in different fertilization treatments based on the data of the sPLS analysis. The red and blue colors indicate positive and negative correlations respectively.

In the PLS-DA of the foliar metabolomes of *T*. *panamensis*, control trees differed most from P and K fertilized trees along component 1 and from K, N and NK fertilized trees along component 2 (Fig 4). P and K fertilized trees had the lowest concentrations of most amino acids and the highest concentrations of nicotine, disacharide and ascorbic acid (vit C) (Fig 4). The cluster image of the metabolites showed significantly different concentrations among treatments in the PLS-DA of *T*. *panamensis*. A cluster of 30 non-identified metabolites had highest concentration in trees fertilized with K (Fig 5), and a cluster of 23 non-identified metabolites had highest concentration in control trees (Fig 5). The greatest hierarchal differences among overall metabolome structures were between controls versus all other fertilization treatments (Fig 5). Overall metabolome structure also differed significantly between K and P fertilized treatments and the remaining treatments (Table 2, Fig 5).

**Fig 6:**
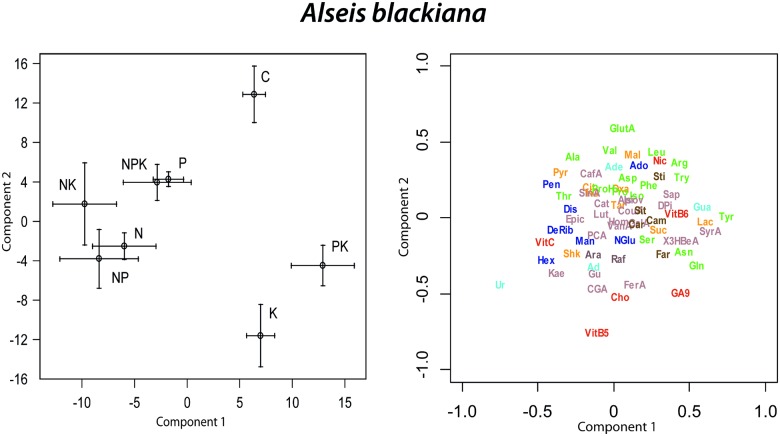
Biplots of the two first components of the PLS-DA of *Alseis blackiana* metabolomics data presenting the mean scores (± 1 S.E.) of the different fertilization treatments (N, P, K, NP, NK, PK, NPK and C) and the metabolomics variables distribution (only the elucidated molecules are depicted). Compounds as in Fig 1.

**Fig 7:**
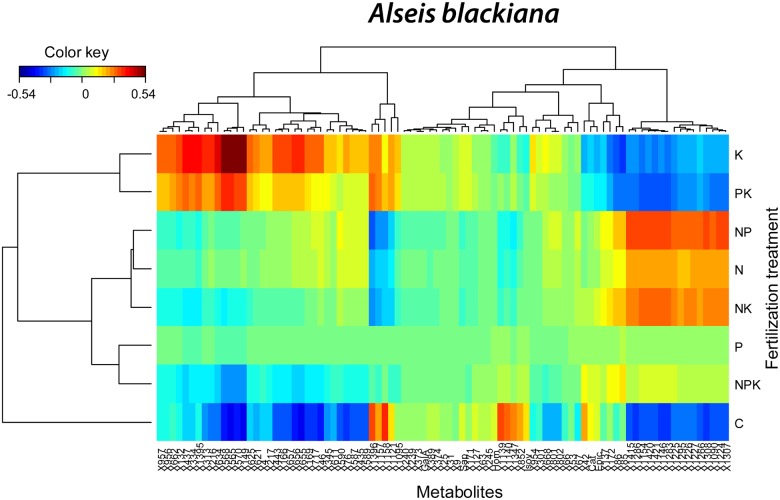
Clustered image maps of the metabolites of *Alseis blackiana* in different fertilization treatments based on the data of the sPLS analysis. The red and blue colors indicate positive and negative correlations respectively.

In the PLS-DA of the foliar metabolomes of *A*. *blackiana*, control trees differed most from N, P, NP, NK and NPK fertilized trees along component 1 and from all fertilized trees along component 2 (Fig 6). The highest concentrations of most identified sugars occurred in K and PK fertilized trees and the lowest concentrations of most identified amino acids occurred in control trees (Fig 6). The cluster image map showed highest concentrations for a cluster of 32 non-identified metabolites in K and PK fertilized trees and lowest concentrations for a second cluster of 23 non-identified metabolites and caffeine and (-)-epicatechin in NP, N and NK fertilized trees (Fig 7). We observed a third cluster of 16 non-identified metabolites that had greater concentrations in N, NK and NP fertilized than in control and P, N, K, PK and NPK fertilized trees (Fig 7). The greatest hierarchical differences among overall metabolome structure were between controls versus all other fertilization treatments (Fig 7). The overall metabolomes of N, NP, NK, P and NPK fertilized *A*. *blackiana* trees were also significantly different than those of K, KP and NP fertilized trees (Figs 6 and 7).

**Fig 8:**
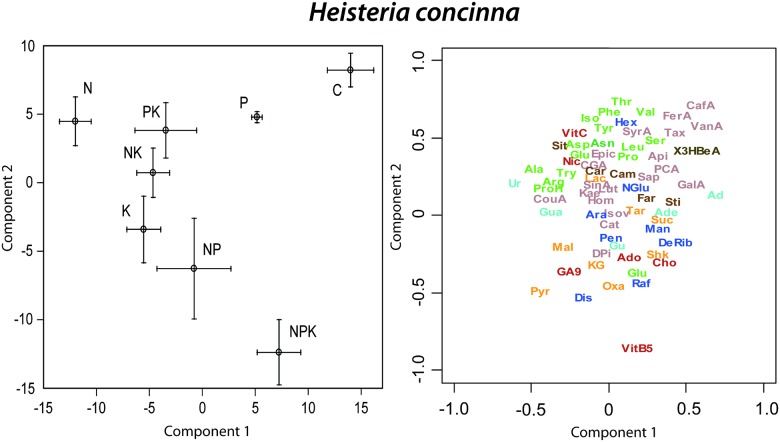
Biplots of the two first components of the PLS-DA of *Heisteria concinna* metabolomics data presenting the mean scores (± 1 S.E.) of the different fertilization treatments (N, P, K, NP, NK, PK, NPK and C) and the metabolomics variables distribution (only the elucidated molecules are depicted). Compounds as in Fig 1.

**Fig 9:**
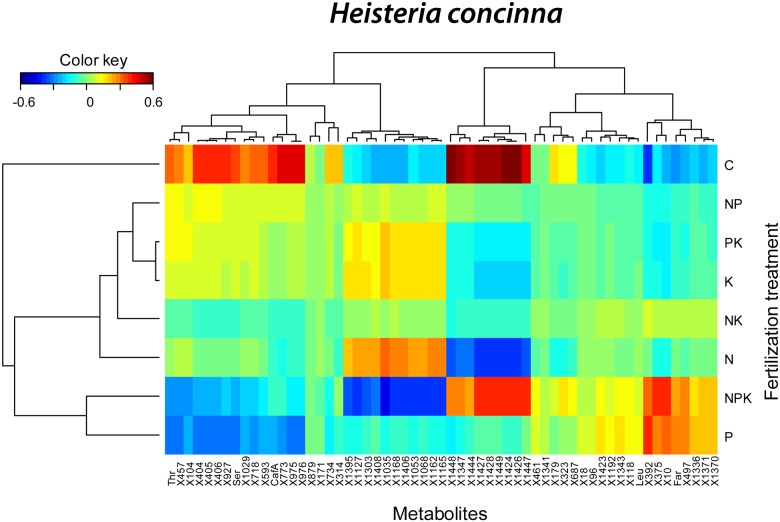
Clustered image maps of the metabolites of *Heisteria concinna* in different fertilization treatments based on the data of the sPLS analysis. The red and blue colors indicate positive and negative correlations respectively.

In the PLS-DA of the foliar metabolomes of *H*. *concinna*, control trees differed most from N, P, K, NP, NK and PK fertilized trees along component 1 and from K, NK, NP and NPK fertilized trees along component 2 (Fig 8). The highest concentrations of several identified secondary metabolites such as caffeine or vainilic acid and the lowest concentrations of several identified amino acids occurred in control trees (Fig 8). Control trees had the highest sugar and organic acid concentrations and the lowest amino acid concentrations (Fig 8). The cluster image map of the 50 metabolites that were able to discriminate among treatments in the sPLS of *H*. *concinna* showed a cluster of 12 non-identified metabolites plus threonine, serine and caffeinic acid with the highest concentration in control trees and the lowest concentrations in P fertilized trees (Fig 9). A second cluster formed by 9 non-identified metabolites had their highest concentrations in N fertilized trees and lowest concentrations in control and NPK fertilized trees (Fig 9). A third cluster of seven non-identified metabolites plus farnesol had their highest concentrations in P and NPK fertilized trees and lowest concentrations in control trees (Fig 9). A final cluster of eleven non-identified metabolites had their highest concentrations in control and NPK fertilized trees and lowest concentrations in N fertilized trees (Fig 9). The greatest hierarchical differences among overall metabolome structure were between controls and all other fertilization treatments (Fig 9). The overall metabolome structures of P and NKP fertilized *H*. *concinna* were also significantly different from the observed metabolomes in the trees growing under the other fertilization treatments (Fig 9).

## Discussion

### Species metabolome

Species differences explained 75% of the total variance of the overall metabolomic data. The three species, *H*. *concinna*, *T*. *panamensis* and *A*. *blackiana* belong to three different orders; Santalales, Sapindales and Gentianales, respectively. These phylogenetic differences are consistent with large interspecific differences in metabolome structure (Fig 1). Foliar metabolomes are the end products of cellular regulatory processes, and their levels can be regarded as the ultimate response of the genotype. The high interspecific variation observed here is consistent with previous studies illustrating different foliar metabolomic profile among plant species [43–47] and even among different genotypes of the same species [45,48,49].

*T*. *panamensis* had the lowest amino acid concentrations among the three studied species and the highest concentrations of most identified phenolics and most identified organic acids such as malate, lactate and pyruvate. These overall results suggest that *T*. *panamensis* up-regulated sugar and phenolics metabolism and down-regulated amino acid synthesis with respect to the other two species studied. The highest concentrations of pyruvate, shikimic acid and of catechin, epicatechin, quercetin and several other polyphenolics in *T*. *panamensis* were consistent with an up-regulation of the shikimic acid pathway. In higher plants, shikimic acid comes from pyruvate and is the precursor of most groups of phenolic compounds such as flavan-3-ols (catechins and epicatechis), flavonols (kaempferol and luteonin) and vanilloids [50,51], all of which were present in higher concentrations in *T*. *panamensis* than in *A*. *blackiana* and *H*. *concinna*. This displacement of primary metabolism to organic acids and thereafter to shikimic acid pathways and C-rich secondary compounds in *T*. *panamensis* is consistent with the lower foliar N concentrations in this species than in *A*. *blackiana* and *H*. *concinna* [21]. Thus despite the analyzed leaves of *T*. *panamensis* were of saplings growing in understory in shade conditions the results showed a probable genotype adaptation of this species which reaches higher adult height and is exposed to more sunlight than the other two species. Plant species adapted to high sunlight frequently have lower foliar N concentrations than plants adapted to more shaded environments [52,53] due to the great environmental capacity to saturate photosynthesis at high light availability [54]. Thus, the higher phenolic concentrations observed in *T*. *panamensis* could be related to the oxidative stresses associated with high irradiance or at high doses of ultraviolet radiation [55–60]. Also, flavones that help attenuate the intensity of light reaching the photosynthetic cells [61] increase in response to high light levels. Furthermore, UV irradiance induces flavonoids (particularly kaempferol derivatives) and isoflavonoids [62–65], UV-absorbing compounds that protect DNA from dimerization and breakage and in general from UV-B damage and subsequent cell death. Moreover, other possible explanations can not be discarded such as that low foliar N concentrations and high phenolics are also anti-herbivore strategies that moreover, may be also consistent with a conservative growth strategy of a shade tolerant sapling in the understory of a closed canopy tropical forest[66].

Differently to *T*. *panamensis*, *A*. *blackiana* had highest concentrations of the amino acids coming from the biosynthesis pathway of glycerate-3P such as serine, and also coming from the *biosynthetic* pathway of oxaloacetate such as asparagine and threonine and also from the biosynthesis pathways of α-ketoglutarate such as proline and glutamine. *A*. *blackiana* also had the highest concentrations of vitamins B6 and nicotinamide, and of some phytosterols such as sitosterol and campesterol and some phenolics such as caffeic acid, 3-hydroxybenzoic acid and D-pinitol. *A*. *blackiana* a canopy medium-size tree presents the highest concentration of the two end-products of the phytosterols synthesis pathway, campesterol and phytosterol [67], both with high capacity to cell membrane protection against oxidative power [68], illustrating that this species had different molecular pathways related to antioxidative strategy than *T*. *panamensis*. It also has a variety of secondary metabolites including non-protein amino acids, flavonoids, flavan-3-ols, saponins and amines that anti-herbivore compounds [69,70].

The subcanopy tree species *H*. *concinna* had the highest concentrations of the amino acids coming from the pyruvate synthesis pathways such as alanine and leucine. *H*. *concinna* had the highest concentrations of vitamin B5, gallic acid and N-acetil-D-glucosamine. In accordance with the predictions, the stimulation of secondary metabolite production has been demonstrated under high light [71,72] or low light; [73,74], thus illustrating that different secondary metabolic pathways can be down- or high- regulated to adapt to both low or high light intensities.

Thus, metabolomic differences among the three studied species in their juvenile stage were consistent with the ecological niche occupied in their adult stage.

### Fertilization effects on metabolomes

Despite the great inter-specific variability and the possible long-term adaptation after 15 years of chronic fertilization treatments, significant metabolome differences were detected among fertilization treatments. The metabolome structure of fertilized trees in all nutrient combinations shifted with respect to the metabolome of control trees (Figs 2a, 3, 4a, 5, 6a, 7, 8a and 9) illustrating an overall shift in metabolome profile in response to changes in nutrient availability.

Notably, differences in overall metabolome structure between control and fertilized trees were species-specific and fertilization treatment-specific as shown in the hierarchical and multivariate analyses. In the PLS-DAs conducted for the three species separately, we observed that *Tetragastris panamensis* the metabolome of the plants with K and P fertilization tend to have the most different metabolome with respect the other fertilization treatments and controls, shifting its metabolome towards higher concentrations of most identified phenolics compounds. The results of the PLS and CIM multivariate analyses were also consistent with the PERMANOVA analyses. Thus, *T*. *panamensis* invested the higher nutrient availability in more antioxidant power making its metabolomic profile even more different than the other to studied species, mainly from *H*. *concinna*.

The univariate analyses show that between 4.1–7.1% (depending on the species) of the individual detected metabolic variables had a significant difference at least between two different fertilization treatments with respect to the controls. Despite the great inter-specific variability and the possible long-term adaptation after 15 years of chronic fertilization treatments, significant metabolome differences were detected among fertilization treatments.

In *Alseis blackiana* the metabolomes of the plants submitted to K, N, NP and NK fertilization tend to have the most different metabolome with respect the other fertilization treatments and controls. The results of the PLS and CIM multivariate analyses were also consistent with the PERMANOVA analyses, illustrating significant overall metabolome shifts due to N fertilization in *A*. *blackiana*. Similarly, *A*. *blackiana*, shifted its metabolome towards higher concentrations of most identified phenolic compounds in most fertilization experiments. The changes in metabolomic profile in response to treatments containing N observed in *A*. *blackiana* could be associated with an enhancement in maximum photosynthetic rates and increases in the plant carbon gain previously observed in this species in response to N fertilization [20]. Moreover, in this medium size canopy species most fertilization treatments led to lower concentrations in most identified amino acids and higher concentrations in most identified sugars and terpenes.

The profile structures of the metabolome of *T*. *panamensis* and *A*. *blackiana* showed a significant shift due to K fertilization. These metabolomic results were consistent with previous studies in these same experimental plots that have observed that these two species respond to K fertilization with changes in root-to-shoot ratio (by ca. + 36%), fine root turnover (by ca.—55%), tissue nutrient concentration (by + 4–8% of K foliar concentration) and also increases of relative growth rate (by approx. + 50% in some years) [12,14,16,20,35]. Moreover, in *Alseis blackiana* P and PK addition increased stomatal conductance and K addition increased quantum yield of photosystem II [20]. These results suggest that K could be limiting for *T*. *panamensis* and *A*. *blackiana* whereas P could be limiting for *A*. *blackiana* reinforcing the idea of the multiple element co-limitation and also the distinct target nutrient limitation for different species in this tropical rainforest.

There were some species-specific trends. In *T*. *panamensis*, fertilization treatments, especially K and P fertilization, shifted metabolome profile towards higher concentrations for most identified phenolic compounds with respect to controls. In this large-size canopy species, K and P fertilization were thus related to antioxidant power provided by the phenolic compounds that is useful in a species receiving high sunlight intensity. Similarly, *A*. *blackiana*, shifted its metabolome towards higher concentrations of most identified phenolic compounds in most fertilization experiments. Conversely, no significant effects of any nutrient fertilization treatment on overall metabolome profile was observed in the PERMANOVA analyses in the sub-canopy species *H*. *concinna*, but the PLS and CIM multivariate analyses were significantly different from controls in N and P. In this species the trees under N, P and NPK fertilization treatments had different overall metabolome structure than those growing under the other fertilization treatments. Particularly N fertilization in *H*. *concinna* shifted metabolomic profile towards higher concentrations for most identified amino acids. This was consistent with the higher foliar N resorption proficiency observed in this species under N fertilization [21]. Thus, the results suggest that this species under higher N availability allocate more N to protein metabolism. This is consistent with plant adaptation to shade conditions by achieving a favorable photosynthesis capacity through a great density of leaf proteins linked to capture of light and to light use-efficiency [75–77].

The data obtained after these years of chronic NPK fertilization has thus provided strong evidence that different nutrients limit species differently depending on the forest strata where they grow, as previously observed for community and ecosystem level variables [12]. In this way, the different shifts in metabolome structure observed in the different studied species were also consistent with the different species-specific ecology strategy and role in this rainforest. Thus, under higher resource availability the two canopy species shifted their metabolome towards large investment on protection mechanisms against sunlight, pathogens, or herbivores, whereas the sub-canopy species followed a different strategy under higher nutrient availability was increasing its primary metabolism and particularly amino acid, further suggesting a rise in its protein metabolism. This result is consistent with the increase of photosynthetic capacity based on higher concentrations of enzymes linked to light capture such as observed in previous studies in response to N fertilization under low light availability [78].

Purine base compounds generally shifted towards higher concentrations in trees under fertilization treatments containing P in *T*. *panamensis* and *H*. *concinna*. This was not observed in *A*. *blackiana*. All these results were consistent with the results of a previous study of foliar elemental analyses illustrating that P fertilization increases foliar P concentration in all three studied species [21]. However, whereas this increase in foliar P concentration was equally due to increases in P_inorganic_ and to P_organic_ in *T*. *panamensis* and *A*. *blackiana*, in *H*. *concinna* was mainly due to P_inorganic_ [21]. Moreover, this relationship between the shifts in metabolomic profile and the changes in elemental composition has been also observed in previous studies [79], and is consistent with the fact that different plant functions need the different bio-elements in different proportions [10,33,80].

P fertilization, but not N+P fertilization, increased foliar P concentration in all three studied species [19]. Consistently with these previous results we have observed that Purine base compounds (rich in P_organic_) generally shifted towards higher concentrations in trees under fertilization treatments containing P in *T*. *panamensis* and *H*. *concinna*, but not in *A*. *blackiana*. Coupling metabolomics data with elemental composition data, the relationship between the shifts in metabolomic profile and the changes in elemental composition has been observed in previous studies [79], being consistent with the fact that different plant functions need the different bio-elements in different proportions [10,33,80]. But further to this, in this current study, we have observed that metabolomic data coupled to elemental composition data allows the observation of how different plant species invest the new sources of one determined element (here P) in different plant functions; *T*. *panamensis* and *H*. *concinna* in molecules involved in growth and energy transfer, whereas in *A*. *blackiana* the extra supply of P provided by fertilization remains mostly as inorganic phosphate in cells.

Thus, our results suggest a species-specific use of the diverse nutrients when their availability changes. All three species have different metabolic profile shift in response to the change in the availability of the three studied elements. The results thus do not suggest a general limiting role of any of three nutrients, but all three nutrients probably limited some functions in some species.

## Conclusions

The metabolomic results show that different species use and are limited by distinct nutrients. The different species-specific metabolome profiles and also the species-specific changes in metabolome structure in response to the fertilization treatments were consistent with the differences in ecological habitats of each species in the rainforest and with most of their ecophysiological changes in growth, physiology or elemental composition observed in other studies of this tropical forest site. They highlight the asymmetrical species-specific use of different nutrients in this highly diverse tropical forest. These results suggest that nutrient imbalances, as for example those due to future atmospheric N deposition, could have asymmetrical species-specific effects with further consequences on species diversity and community structure. The study provides a further evidence of the great sensitivity of ecometabolomic profile studies for detecting genotypic differences. Despite the great inter-specific variability and the possible long-term adaptation to fertilization treatments, ecometabolomic profile study detected significant metabolome profile differences among different fertilization treatments.

## Supporting information

S1 Table The full dataset with all of identified metabolomics and unknowns variables provident of MS raw data in leaf of three species in different fertilization treatments. The units of the variables are intensity of the value of deconvoluted total intensities.
(DOCX)

S2 Table One way ANOVA for the PLS-DA scores of the fertilization treatments. Bold type indicates significant effects (*P* < 0.05).
(DOCX)

S3 Table Post-hoc Bonferroni tests from the one way ANOVA show in S1 Table for all pairwise comparisons of the PC1 scores of the PLS-DA analysis of fertilization treatments and control. Bold type indicates significant effects (*P* < 0.05) Italics type indicates marginally significant effects (*P* < 0.1).
(DOCX)

## References

[1] Fernández-Martínez M, Vicca S, Janssens I a., Sardans J, Luyssaert S, Campioli M, et al Nutrient availability as the key regulator of global forest carbon balance. Nat Clim Chang. 2014;4: 471–476.

[2] Walker TW, Syers JK. The fate of phosphorus during pedogenesis. Geoderma. 1976;15: 1–19.

[3] Vitousek PM, Porder S, Houlton BZ, Chadwick O a. Terrestrial phosphorus limitation: Mechanisms, implications, and nitrogen-phosphorus interactions. Ecol Appl. 2010;20: 5–15. 20349827 10.1890/08-0127.1

[4] Hedin LO, Vitousek PM, Matson P a. Nutrient Losses Over Four Million Years of Tropical Forest Development. Ecology. 2003;84: 2231–2255.

[5] Lambers H, Raven J a, Shaver GR, Smith SE. Plant nutrient-acquisition strategies change with soil age. Trends Ecol Evol. 2008;23: 95–103. 10.1016/j.tree.2007.10.008 18191280

[6] Vitousek PM. Nutrient cycling and limitation: Hawai ‘i as a model system. Princeton. Princeton, New Jersey, USA.; 2004.

[7] Tripler CE, Kaushal SS, Likens GE, Walter MT. Patterns in potassium dynamics in forest ecosystems. Ecol Lett. 2006;9: 451–466. 10.1111/j.1461-0248.2006.00891.x 16623731

[8] LeBauer DS, Treseder KK. Nitrogen limitation of net primary productivity in terrestrial ecosystems is globally distributed. Ecology. 2008;89: 371–9. 18409427 10.1890/06-2057.1

[9] Elser JJ, Bracken MES, Cleland EE, Gruner DS, Harpole WS, Hillebrand H, et al Global analysis of nitrogen and phosphorus limitation of primary producers in freshwater, marine and terrestrial ecosystems. Ecol Lett. 2007;10: 1135–1142. 10.1111/j.1461-0248.2007.01113.x 17922835

[10] Sardans J, Peñuelas J. Potassium: a neglected nutrient in global change. Glob Ecol Biogeogr. 2015; 261–275.

[11] Paoli GD, Curran LM. Soil nutrients limit fine litter production and tree growth in mature lowland forest of southwestern Borneo. Ecosystems. 2007;10: 503–518.

[12] Wright SJ, Yavitt JB, Wurzburger N, Turner BL, Tanner EVJ, Sayer EJ, et al Potassium, phosphorus, or nitrogen limit root allocation, tree growth, or litter production in a lowland tropical forest. Ecology. 2011;92: 1616–25. Available: http://www.ncbi.nlm.nih.gov/pubmed/21905428 21905428 10.1890/10-1558.1

[13] Santiago LS. Nutrient limitation of eco-physiological processes in tropical trees. Trees. Springer Berlin Heidelberg; 2015;29: 1291–1300.

[14] Santiago LS, Wright SJ, Harms KE, Yavitt JB, Korine C, Garcia MN, et al Tropical tree seedling growth responses to nitrogen, phosphorus and potassium addition. J Ecol. 2012;100: 309–316.

[15] Pasquini SC, Santiago LS. Nutrients limit photosynthesis in seedlings of a lowland tropical forest tree species. Oecologia. 2012;168: 311–319. 10.1007/s00442-011-2099-5 21837408

[16] Pasquini SC, Wright SJ, Santiago LS. Lianas always outperform tree seedlings regardless of soil nutrients: results from a long-term fertilization experiment. Ecology. 2014;96: 141217123415005. 10.1890/14-1660.1 26378309

[17] Wurzburger N, Wright SJ. Fine-root responses to fertilization reveal multiple nutrient limitation in a lowland tropical forest. Ecology. 2015;96: 2137–2146. 26405739 10.1890/14-1362.1

[18] Turner BL, Haygarth PM. Phosphatase activity in temperate pasture soils: Potential regulation of labile organic phosphorus turnover by phosphodiesterase activity. Sci Total Environ. 2005;344: 27–36. 10.1016/j.scitotenv.2005.02.003 15907508

[19] Yavitt JB, Harms KE, Garcia MN, Mirabello MJ, Wright SJ. Soil fertility and fine root dynamics in response to 4 years of nutrient (N, P, K) fertilization in a lowland tropical moist forest, Panama. Austral Ecol. 2011;36: 433–445.

[20] Pasquini SC, Santiago LS. Nutrients limit photosynthesis in seedlings of a lowland tropical forest tree species. Oecologia. 2012;168: 311–319. 10.1007/s00442-011-2099-5 21837408

[21] Mayor JR, Wright SJ, Turner BL. Species-specific responses of foliar nutrients to long-term nitrogen and phosphorus additions in a lowland tropical forest. J Ecol. 2014;102: 36–44.

[22] Peñuelas J, Sardans J, Llusià J, Owen SM, Carnicer J, Giambelluca TW, et al Faster returns on “leaf economics” and different biogeochemical niche in invasive compared with native plant species. Glob Chang Biol. 2009;16: 2171–2185.

[23] Peñuelas J, Sardans J, Ogaya R, Estiarte M. Nutrient stoichiometric relations and biogeochemical niche in coexisting plant species: Effect of simulated climate change. Polish J Ecol. 2008;56: 613–622.

[24] Sardans J, Janssens IA, Alonso R, Veresoglou SD, Rillig MC, Sanders TG, et al Foliar elemental composition of European forest tree species associated with evolutionary traits and present environmental and competitive conditions. Glob Ecol Biogeogr. 2015;24: 240–255.

[25] Sardans J, Peñuelas J. Tree growth changes with climate and forest type are associated with relative allocation of nutrients, especially phosphorus, to leaves and wood. Glob Ecol Biogeogr. 2013;22: 494–507.

[26] Sardans J, Peñuelas J. Hydraulic redistribution by plants and nutrient stoichiometry: Shifts under global change. Ecohydrology. 2014;7: 1–20.

[27] Urbina I, Sardans J, Beierkuhnlein C, Jentsch A, Backhaus S, Grant K, et al Shifts in the elemental composition of plants during a very severe drought. Environ Exp Bot. 2015;111: 63–73. 10.1016/j.envexpbot.2014.10.005 PMC6485511 31031453

[28] Yu Q, Chen Q, Elser JJ, He N, Wu H, Zhang G, et al Linking stoichiometric homoeostasis with ecosystem structure, functioning and stability. Ecol Lett. 2010;13: 1390–1399. 10.1111/j.1461-0248.2010.01532.x 20849443

[29] Fiehn O, Kopka J, Dörmann P, Altmann T, Trethewey RN, Willmitzer L. Metabolite profiling for plant functional genomics. Nat Biotechnol. 2000;18: 1157–61. 10.1038/81137 11062433

[30] Peñuelas J, Sardans J. Ecological metabolomics. Chem Ecol. 2009;25: 305–309.

[31] Leiss KA, Choi YH, Verpoorte R, Klinkhamer PGL. An overview of NMR-based metabolomics to identify secondary plant compounds involved in host plant resistance. Phytochem Rev. 2011;10: 205–216. 10.1007/s11101-010-9175-z 21765818 PMC3105236

[32] Gargallo-Garriga A, Sardans J, Pérez-Trujillo M, Rivas-Ubach A, Oravec M, Vecerova K, et al Opposite metabolic responses of shoots and roots to drought. Sci Rep. 2014;4: 1–7. 10.1038/srep06829 PMC4212232 25351427

[33] Peñuelas J, Sardans J. Ecology: Elementary factors. Nature. 2009;460: 803–804. 10.1038/460803a 19675634

[34] Fiehn O. Metabolomics—the link between genotypes and phenotypes. Plant Mol Biol. 2002;48: 155–71. Available: http://www.ncbi.nlm.nih.gov/pubmed/11860207 11860207

[35] Yavitt JB, Harms KE, Garcia MN, Wright SJ, He F, Mirabello MJ. Spatial heterogeneity of soil chemical properties in a lowland tropical moist forest, Panama. Aust J Soil Res. 2009;47: 674.

[36] Wilson M, Lindow SE. Coexistence among Epiphytic Bacterial Populations Mediated through Nutritional Resource Partitioning. Appl Environ Microbiol. 1994;60: 4468–4477. 16349462 10.1128/aem.60.12.4468-4477.1994 PMC202007

[37] Binkley D, Giardina C, Bashkin MA. Soil phosphorus pools and supply under the influence of Eucalyptus saligna and nitrogen-fixing Albizia facaltaria. For Ecol Manage. 2000;128: 241–247.

[38] Mirmanto E, Proctor J, Green J, Nagy L. Effects of nitrogen and phosphorus fertilization in a lowland evergreen rainforest. Philos Trans R Soc London B Biol Sci. 1999;354: 1825–1829. 10.1098/rstb.1999.0524 11605625 PMC1692691

[39] Tanner EVJ, Kapos V, Franco W. Nitorgen and Phosphorus Fertilization Effects on Venezuelan Montane Forest Trunk Growth and Litterfall. Ecology. 1992;73: 78–86.

[40] Corre MD, Veldkamp E, Arnold J, Wright SJ. Impact of elevated N input on soil N cycling and losses in old-growth lowland and montane forests in Panama. Ecology. 2010;91: 1715–1729. 20583713 10.1890/09-0274.1

[41] Gargallo-Garriga A, Sardans J, Pérez-Trujillo M, Guenther A, Llusià J, Rico L, et al Shifts in plant foliar and floral metabolomes in response to the suppression of the associated microbiota. BMC Plant Biol. BMC Plant Biology; 2016;16: 78 10.1186/s12870-016-0767-7 27048394 PMC4822282

[42] Gargallo-Garriga A, Sardans J, Pérez-Trujillo M, Oravec M, Urban O, Jentsch A, et al Warming differentially influences the effects of drought on stoichiometry and metabolomics in shoots and roots. New Phytol. 2015;207: 591–603. 10.1111/nph.13377 25772030

[43] Bernhardsson C, Robinson KM, Abreu IN, Jansson S, Albrectsen BR, Ingvarsson PK. Geographic structure in metabolome and herbivore community co-occurs with genetic structure in plant defence genes. Ecol Lett. 2013;16: 791–798. 10.1111/ele.12114 23601188

[44] Carreno-Quintero N, Bouwmeester HJ, Keurentjes JJB. Genetic analysis of metabolome-phenotype interactions: From model to crop species. Trends Genet. 2013;29: 41–50. 10.1016/j.tig.2012.09.006 23084137

[45] Montero-Vargas JM, González-González LH, Gálvez-Ponce E, Ramírez-Chávez E, Molina-Torres J, Chagolla A, et al Metabolic phenotyping for the classification of coffee trees and the exploration of selection markers. Mol Biosyst. 2013;9: 693–9. 10.1039/c3mb25509c 23385826

[46] Schweiger R, Baier MC, Perscke M, Muller C. Hugh specificity in plant leaf metabolomics responses to arbuscular mycorrhiza. Nat Commun. 2014;5: 3886 10.1038/ncomms4886 24848943

[47] Tohge T, de Souza LP, Fernie AR. Genome-enabled plant metabolomics. J Chromatogr B Anal Technol Biomed Life Sci. Elsevier B.V.; 2014;966: 7–20. 10.1016/j.jchromb.2014.04.003 24811977

[48] Robinson AR, Gheneim R, Kozak R a, Ellis DD, Mansfield SD. The potential of metabolite profiling as a selection tool for genotype discrimination in Populus. J Exp Bot. 2005;56: 2807–2819. 10.1093/jxb/eri273 16143717

[49] Eckert AJ, Wegrzyn JL, Cumbie WP, Goldfarb B, Huber D a, Tolstikov V, et al Association genetics of the loblolly pine (Pinus taeda, Pinaceae) metabolome. New Phytol. 2012;193: 890–902. 10.1111/j.1469-8137.2011.03976.x 22129444

[50] Ghasemzadeh A, Ghasemzadeh N. Flavonoids and phenolic acids: Role and biochemical activity in plants and human. J Med Plants Res. 2011;5: 6697–6703.

[51] Ameh SJ, Tarfa FD, Ebeshi BU. Traditional herbal management of sickle cell anemia: Lessons from Nigeria. Anemia. 2012;2012. 10.1155/2012/607436 PMC3502758 23198140

[52] Niinemets U. Role of foliar nitrogen in light harvesting and shade tolerance of four temperate deciduous woody species. Funct Ecol. 1997;11: 518–531.

[53] Kitajima K. depend tree seedlings tropical Do shade-tolerant analysis growth Functional longer on seed reserves? species of three Bignoniaceae. Funct Ecol. 2002;16: 433–444.

[54] Marino G, Aqil M, Shipley B. The leaf economics spectrum and the prediction of photosynthetic light-response curves. Funct Ecol. 2010;24: 263–272.

[55] Cheynier V, Comte G, Davies KM, Lattanzio V, Martens S. Plant phenolics: Recent advances on their biosynthesis, genetics, andecophysiology. Plant Physiol Biochem. Elsevier Masson SAS; 2013;72: 1–20. 10.1016/j.plaphy.2013.05.009 23774057

[56] Estiarte M, Peñuelas J. Excess carbon: the relationship with phenotypical plasticity in storage and defense functions of plants. Orsis Org i Sist Rev botànica Zool i Ecol. 1999;14: 159–203. Available: http://dialnet.unirioja.es/servlet/articulo?codigo=809419&info=resumen

[57] Klem K, Holub P, Štroch M, Nezval J, Špunda V, Tříska J, et al Ultraviolet and photosynthetically active radiation can both induce photoprotective capacity allowing barley to overcome high radiation stress. Plant Physiol Biochem. 2015;93: 74–83. 10.1016/j.plaphy.2015.01.001 25583309

[58] Martz F, Jaakola L, Julkunen-Tiitto R, Stark S. Phenolic Composition and Antioxidant Capacity of Bilberry (Vaccinium myrtillus) Leaves in Northern Europe Following Foliar Development and Along Environmental Gradients. J Chem Ecol. 2010;36: 1017–1028. 10.1007/s10886-010-9836-9 20721607

[59] Zhao HJ, Zou Q. Protective effects of exogenous antioxidants and phenolic compounds on photosynthesis of wheat leaves under high irradiance and oxidative stress. Photosynthetica. 2002;40: 523–527.

[60] Randi A, Freitas M, Rodrigues A, Maraschin M, Torres M. Acclimation and photoprotection of young gametophytes of Acrostichum danaeifolium to UV-B stress. Photosynthetica. 2014;52: 50–56.

[61] Beggs JJ, Chapman BJ. Australian Strike Activity in an International Context: 1964–85. J Ind Relations. 1986;2: 137–149.

[62] Hahlbrock K, Lamb CJ, Purwin C, Ebel J, Fautz E, Schäfer E. Rapid Response of Suspension-cultured Parsley Cells to the Elicitor from Phytophthora megasperma var. sojae INDUCTION OF THE ENZYMES OF GENERAL PHENYLPROPANOID METABOLISM. Plant Physiol. 1981;67: 768–773. 16661752 10.1104/pp.67.4.768 PMC425770

[63] Beggs CJ, Stolzer-Jehle A, Wellmann E. Isoflavonoid formation as an indicator of UV stress in bean (Phaseolus vulgaris L.) leaves. Plant Physiol. 1985;79: 630–634. 16664463 10.1104/pp.79.3.630 PMC1074942

[64] Li J, Ou-Lee TM, Raba R, Amundson RG, Last RL. Arabidopsis Flavonoid Mutants Are Hypersensitive to UV-B Irradiation. Plant Cell. 1993;5: 171–179. 10.1105/tpc.5.2.171 12271060 PMC160260

[65] Lois R. Accumulation of UV-absorbing flavonoids induced by UV-B radiation inAmbidopsis thaliana L. Planta. 1994;194: 498–503.

[66] Stamp N. Out of the quagmire of lant defense hypotheses. Q Rev Biol. 2003;78: 23–55. 12661508 10.1086/367580

[67] Nguyen HTM, Neelakadan AK, Quach TN, Valliyodan B, Kumar R, Zhang Z, et al Molecular characterization of Glycine max squalene synthase genes in seed phytosterol biosynthesis. Plant Physiol Biochem. Elsevier Masson SAS; 2013;73: 23–32. 10.1016/j.plaphy.2013.07.018 24036394

[68] Gil M, Pontin M, Berli F, Bottini R, Piccoli P. Metabolism of terpenes in the response of grape (Vitis vinifera L.) leaf tissues to UV-B radiation. Phytochemistry. Elsevier Ltd; 2012;77: 89–98. 10.1016/j.phytochem.2011.12.011 22277733

[69] Lokvam J, Kursar TA. Divergence in structure and activity of phenolic defenses in young leaves of two co-occurring Inga species. J Chem Ecol. 2005;31: 2563–2580. 10.1007/s10886-005-7614-x 16273429

[70] Lokvam J, Coley PD, Kursar TA. Cinnamoyl glucosides of catechin and dimeric procyanidins from young leaves of Inga umbellifera (Fabaceae). Phytochemistry. 2004;65: 351–358. 14751307 10.1016/j.phytochem.2003.11.012

[71] Skillman JB, Garcia M, Virgo A, Winter K. Growth irradiance effects on photosynthesis and growth in two co-occurring shade-tolerant neotropical perennials of contrasting photosynthetic pathways. Am J Bot. 2005;92: 1811–1819. 10.3732/ajb.92.11.1811 21646098

[72] Gyimah R, Nakao T. Early growth and photosynthetic responses to light in seedlings of three tropical species differing in successional strategies. New For. 2007;33: 217–236.

[73] Zavala JA, Ravetta DA. Allocation of photoassimilates to biomass, resin and carbohydrates in Grindelia chiloensis as affected by light intensity. F Crop Res. 2001;69: 143–149.

[74] Coelho GC, Rachwal MFG, Dedecek RA, Curcio GR, Nietsche K, Schenkel EP. Effect of light intensity on methylxanthine contents of Ilex paraguariensis A. St. Hil. Biochem Syst Ecol. 2007;35: 75–80.

[75] Gianoli E, Saldaña A. Phenotypic selection on leaf functional traits of two congeneric species in a temperate rainforest is consistent with their shade tolerance. Oecologia. 2013;173: 13–21. 10.1007/s00442-013-2590-2 23334233

[76] Tang J, Baldocchi DD, Xu L. Tree photosynthesis modulates soil respiration on a diurnal time scale. Glob Chang Biol. 2005;11: 1298–1304.

[77] Wang WQ, Sardans J, Wang C, Zeng CS, Tong C, Asensio D, et al Ecological stoichiometry of C, N, and P of invasive Phragmites australis and native Cyperus malaccensis species in the Minjiang River tidal estuarine wetlands of China. Plant Ecol. 2015; 809–822.

[78] Grassi G, Minotta G. Influence of nutrient supply on shade-sun acclimation of Picea abies seedlings: effects on foliar morphology, photosynthetic performance and growth. Tree Physiol. 2000;20: 645–652. 12651514 10.1093/treephys/20.10.645

[79] Rivas-ubach A, Sardans J, Pérez-Trujillo M, Estiarte M, Peñuelas J. Strong relationship between elemental stoichiometry and metabolome in plants. Proc Natl Acad Sci U S A. 2012;109: 4181–6. 10.1073/pnas.1116092109 22371578 PMC3306711

[80] Sardans J, Peñuelas J. Climate and taxonomy underlie different elemental concentrations and stoichiometries of forest species: the optimum “biogeochemical niche.” Plant Ecol. 2014;215: 441–455. 10.1007/s11258-014-0314-2 25983614 PMC4430814

