# Supplementary material for: Long-term fertilization determines different metabolomic profiles and responses in saplings of three rainforest tree species with different adult canopy position
Source: PLoS One. 2017 May 11;12(5):e0177030. doi: 10.1371/journal.pone.0177030 (PMC5426662; doi:10.1371/journal.pone.0177030)
Supplement: S1 Table — The units of the variables are intensity of the value of deconvoluted total intensities. (DOCX) [file pone.0177030.s001.docx]

**S1 Table**. The full dataset with all of identified metabolomics and unknowns variables provident of MS raw data in leaf of three species in different fertilization treatments. The units of the variables are intensity of the value of deconvoluted total intensities.
