# Supplementary material for: Long-term fertilization determines different metabolomic profiles and responses in saplings of three rainforest tree species with different adult canopy position
Source: PLoS One. 2017 May 11;12(5):e0177030. doi: 10.1371/journal.pone.0177030 (PMC5426662; doi:10.1371/journal.pone.0177030)
Supplement: S2 Table — Bold type indicates significant effects (P < 0.05). (DOCX) [file pone.0177030.s002.docx]

**S2 Table.** One way ANOVA for the PLS-DA scores of the fertilization treatments. Bold type indicates significant effects (*P* < 0.05).

|  | **Degr. of Freedom** | **SS** | **MS** | **F** | **P** |
| --- | --- | --- | --- | --- | --- |
| **Intercept** | 1 | 2.424 | 2.4239 | 0.039436 | 0.843085 |
| **Fertilization treatment** | **7** | **1741.755** | **248.8221** | **4.048250** | **0.000751** |
| **Error** | 81 | 4978.594 | 61.4641 |  |  |
| **Total** | 88 | 6720.348 |  |  |  |
