# Supplementary material for: Long-term fertilization determines different metabolomic profiles and responses in saplings of three rainforest tree species with different adult canopy position
Source: PLoS One. 2017 May 11;12(5):e0177030. doi: 10.1371/journal.pone.0177030 (PMC5426662; doi:10.1371/journal.pone.0177030)
Supplement: S3 Table — Bold type indicates significant effects (P < 0.05) Italics type indicates marginally significant effects (P < 0.1). (DOCX) [file pone.0177030.s003.docx]

**S3 Table.** Post-hoc Bonferroni tests from the one way ANOVA show in Table S1 for all pairwise comparisons of the PC1 scores of the PLS-DA analysis of fertilization treatments and control. Bold type indicates significant effects (*P* < 0.05) Italics type indicates marginally significant effects (*P* < 0.1).

| Independent variables | P | K | NK | N | NP | C | PK | NPK |
| --- | --- | --- | --- | --- | --- | --- | --- | --- |
| P |  | 0.917087 | 0.643301 | *0.058422* | 0.493269 | **0.001127** | 0.358498 | 0.598902 |
| K | 0.917087 |  | 0.537959 | **0.031682** | 0.394425 | **0.000319** | 0.379282 | 0.509181 |
| NK | 0.643301 | 0.537959 |  | 0.111064 | 0.799678 | **0.001865** | 0.136164 | 0.902839 |
| N | *0.058422* | **0.031682** | 0.111064 |  | 0.187223 | 0.119297 | **0.003358** | 0.199892 |
| NP | 0.493269 | 0.394425 | 0.799678 | 0.187223 |  | **0.004740** | *0.088993* | 0.918424 |
| C | **0.001127** | **0.000319** | **0.001865** | 0.119297 | **0.004740** |  | **0.000018** | **0.008426** |
| PK | 0.358498 | 0.379282 | 0.136164 | **0.003358** | *0.088993* | **0.000018** |  | 0.152176 |
| NPK | 0.598902 | 0.509181 | 0.902839 | 0.199892 | 0.918424 | **0.008426** | 0.152176 |  |
